# Marked improvements in glycaemic outcomes following insulin pump therapy initiation in people with type 1 diabetes: a nationwide observational study in Scotland

**DOI:** 10.1007/s00125-021-05413-7

**Published:** 2021-03-08

**Authors:** Anita Jeyam, Fraser W. Gibb, John A. McKnight, Brian Kennon, Joseph E. O’Reilly, Thomas M. Caparrotta, Andreas Höhn, Stuart J. McGurnaghan, Luke A. K. Blackbourn, Sara Hatam, Rory J. McCrimmon, Graham Leese, Robert S. Lindsay, John Petrie, John Chalmers, Sam Philip, Sarah H. Wild, Naveed Sattar, Paul M. McKeigue, Helen M. Colhoun, On behalf of the Scottish Diabetes Research Network (SDRN) Epidemiology Group

**Affiliations:** 1 MRC Institute of Genetic and Molecular Medicine, University of Edinburgh, Edinburgh, UK; 2 Royal Infirmary of Edinburgh, Edinburgh Centre for Endocrinology and Diabetes, Edinburgh, UK; 3 Western General Hospital, NHS Lothian, Edinburgh, UK; 4 Queen Elizabeth University Hospital, Glasgow, UK; 5 Division of Molecular and Clinical Medicine, University of Dundee, Dundee, UK; 6 Ninewells Hospital, Dundee, UK; 7 Institute of Cardiovascular and Medical Sciences, University of Glasgow, Glasgow, UK; 8 Diabetes Centre, Victoria Hospital, Kirkcaldy, UK; 9 Grampian Diabetes Research Unit, Diabetes Centre, Aberdeen Royal Infirmary, Aberdeen, UK; 10 Usher Institute of Population Health Sciences and Informatics, Centre for Population Health Sciences, School of Molecular, Genetic and Population Health Sciences, University of Edinburgh, Edinburgh, UK; 11 Public Health, NHS Fife, Kirkcaldy, UK

**Keywords:** Diabetes mellitus type 1, HbA_1c_, Hypoglycaemia, Insulin pump, Ketoacidosis

## Abstract

**Aims/hypothesis:**

Our aim was to assess the use of continuous subcutaneous insulin infusion (CSII) in people with type 1 diabetes in Scotland and its association with glycaemic control, as measured by HbA_1c_ levels, frequency of diabetic ketoacidosis (DKA) and severe hospitalised hypoglycaemia (SHH), overall and stratified by baseline HbA_1c_.

**Methods:**

We included 4684 individuals with type 1 diabetes from the national Scottish register, who commenced CSII between 2004 and 2019. We presented crude within-person differences from baseline HbA_1c_ over time since initiation, crude DKA and SHH event-rates pre-/post-CSII exposure. We then used mixed models to assess the significance of CSII exposure, taking into account: (1) the diffuse nature of the intervention (i.e. structured education often precedes initiation); (2) repeated within-person measurements; and (3) background time-trends occurring pre-intervention.

**Results:**

HbA_1c_ decreased after CSII initiation, with a median within-person change of −5.5 mmol/mol (IQR −12.0, 0.0) (−0.5% [IQR −1.1, 0.0]). Within-person changes were most substantial in those with the highest baseline HbA_1c_, with median −21.0 mmol/mol (−30.0, −11.0) (−1.9% [−2.7, −1.0]) change in those with a baseline >84 mmol/mol (9.8%) within a year of exposure, that was sustained: −19.0 mmol/mol (−27.6, −6.5) (−1.7% [−2.5, −0.6]) at ≥5 years. Statistical significance and magnitude of change were supported by the mixed models results. The crude DKA event-rate was significantly lower in post-CSII person-time compared with pre-CSII person-time: 49.6 events (95% CI 46.3, 53.1) per 1000 person-years vs 67.9 (64.1, 71.9); rate ratio from Bayesian mixed models adjusting for pre-exposure trend: 0.61 (95% credible interval [CrI] 0.47, 0.77; posterior probability of reduction pp = 1.00). The crude overall SHH event-rate in post-CSII vs pre-CSII person-time was also lower: 17.8 events (95% CI 15.8, 19.9) per 1000 person-years post-exposure vs 25.8 (23.5, 28.3) pre-exposure; rate ratio from Bayesian mixed models adjusting for pre-exposure trend: 0.67 (95% CrI 0.45, 1.01; pp = 0.97).

**Conclusions/interpretation:**

CSII therapy was associated with marked falls in HbA_1c_ especially in those with high baseline HbA_1c_. CSII was independently associated with reduced DKA and SHH rates. CSII appears to be an effective option for intensive insulin therapy in people with diabetes for improving suboptimal glycaemic control.

**Graphical abstract:**

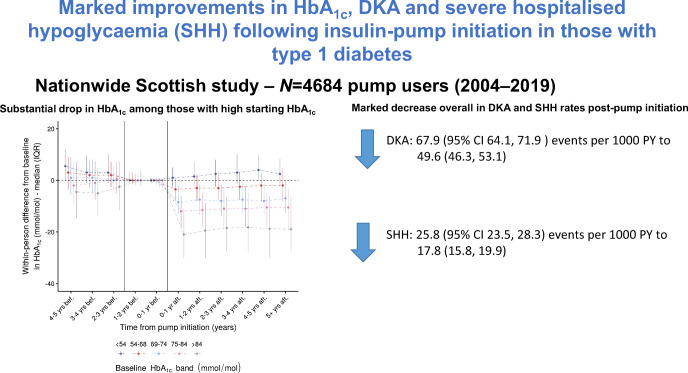

**Supplementary Information:**

The online version contains peer-reviewed but unedited supplementary material available at 10.1007/s00125-021-05413-7.

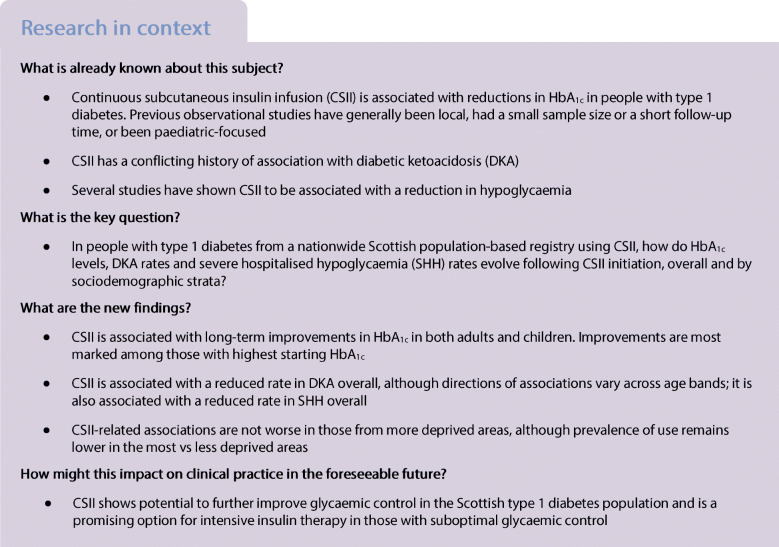



## Introduction

As continuous subcutaneous insulin infusion (CSII) usage to aid diabetes management becomes more widespread, there is still uncertainty regarding its effectiveness and safety. RCTs reported small improvements in HbA_1c_ post-CSII initiation; before/after studies suggested larger effects [1]. Several studies reported a reduction in severe hypoglycaemia rates [1–3]. The effect of CSII on diabetic ketoacidosis (DKA) is unclear as studies conflict in direction of effect reported [2–4].

Criteria for CSII initiation differ across countries [5]. CSII therapy initiation in Scotland follows criteria set by the National Institute for Health and Care Excellence (NICE) guidelines [6], Scottish Government policy and individual National Health Service (NHS) health board budget allocation decisions. A recent study [7] examined the effect of CSII therapy on HbA_1c_ among people with type 1 diabetes in the Scottish health board Lothian and found a significant decrease in HbA_1c_ following CSII initiation, particularly among those with suboptimal glycaemic control. In this paper, we widen the scope of that analysis by focusing on CSII therapy initiation in people with type 1 diabetes throughout Scotland.

Our aim was to: (1) describe the current prevalence of ever-CSII use; and (2) assess the effect of CSII therapy on glycaemic control as measured by HbA_1c_, DKA and severe hospitalised hypoglycaemia (SHH). We furthermore explored differences in CSII therapy effect across baseline HbA_1c_ bands and sociodemographic strata (age band at CSII initiation, sex, area-level deprivation).

## Methods

### Ethics permission

Data and data linkage were originally set up with approval from the Scottish A Research Ethics Committee (ref 11/AL/0225), Caldicott Guardians and the Privacy Advisory Committee (PAC - ref. 33/11), now running with approval from the Public Benefit and Privacy Panel for Health and Social Care (PBPP - reference 1617-0147).

### Data sources

We used anonymised data from the Scottish Care Information – Diabetes Collaboration database (SCI-Diabetes), which holds extensive electronic healthcare records of >99% of people with diabetes in Scotland, both adults and children, since 2004. SCI-Diabetes covers extensive clinical and prescription information including CSII start dates. These data are linked to hospital admissions records and mortality data from the Information Services Division (ISD) of NHS Scotland and the National Records of Scotland. SCI-Diabetes and associated linkage have previously been described [8, 9].

### Study population

Among those alive and observable with type 1 diabetes in SCI-Diabetes at any time from 2004 to 2019, we analysed glycaemic outcomes among those who initiated CSII therapy before June 2019. Type of diabetes was assessed from our validated algorithm [10]. Individuals contributed person-time from the latest of: 1 January 2004, first observability date (based on prescribing, hospital admission or clinical measurements) or date of diabetes diagnosis. Person-time was censored at the earliest of: 12 December 2019 (administrative censoring), date of death, last date of observability, first stop date of CSII (if no restart within a year). Person-time was additionally censored when starting flash monitoring or continuous glucose monitoring (CGM) device after CSII to isolate the effect of CSII initiation from that of other technologies (see electronic supplementary material [ESM] Methods).

### Exposure, outcomes and covariates

Exposure of interest is CSII therapy. Since CSII users in Scotland are selected based on criteria that are not measurable in our data (e.g. motivation, perceived ability to use a pump), we chose to conduct analyses within CSII users. Glycaemic outcomes were compared among CSII users before and after CSII initiation, using data up to a maximum of 5 years prior to initiation. We used a 1:1 non-user control group, matched by age, sex and diabetes duration (ESM Methods) to check for any trend occurring in HbA_1c_ among non-users in similar calendar time, and to inform results obtained in adolescents given the deterioration in glycaemic control in this age group [2, 11].

We defined DKA and SHH as any hospital admission or death involving the ICD-10 (http://apps.who.int/classifications/icd10/browse/2016/en) codes detailed in ESM Methods. Each unique hospital admission was considered as an event. Person-time for DKA/SHH analyses was censored at the last date of available data for hospital admissions: 30 April 2019.

Demographic and clinical information were obtained from SCI-Diabetes. For clinical covariates, baseline values were defined as median value over the 2 years prior to device initiation for continuous variables or the most clinically severe status over this time-window for categorical variables. We used Scottish Index of Multiple Deprivation (SIMD) quintiles as a measure of area-level deprivation, quintile (Q)1 being the most deprived. Age at CSII initiation (years) was categorised into six age bands: <13, ≥13–≤18, ≥19–≤24, ≥25–≤44, ≥45–≤64 and >64; and baseline HbA_1c_ levels (mmol/mol) into five groups, representative of different glycaemic control levels: <54 (7.1%), ≥54–≤68 (8.4%), ≥69–≤74 (8.5–8.9%), ≥75–≤84 (9.0–9.8%) and >84.

### Statistical analysis

All analyses were conducted using R version 3.6.0–64 bit [12] at significance level *p* = 0.05. Missing data were not imputed.

We presented the crude prevalence of ever-CSII users overall and stratified by baseline variable. In order to study the effect of CSII on HbA_1c_, we divided observation time into blocks of 1 year periods before and after CSII initiation, centred on the date of CSII initiation [7], and compared the median HbA_1c_ value of each individual in each time-block. HbA_1c_ levels and absolute within-person differences with respect to baseline were described before and after CSII initiation, overall and stratified by age, baseline HbA_1c_, sex, SIMD and prior flash monitoring/CGM use. Significance of within-person differences were assessed at each time point after CSII initiation, using a one-sided Wilcoxon signed-rank test; *p* values were adjusted for multiple comparison using Bonferroni’s correction.

Crude pre-/post-exposure changes can under/overestimate an intervention’s effect when there is a pre-intervention trend in the outcome which is not taken into account. Log-transformed HbA_1c_ was modelled using linear mixed models [13] to assess whether changes were similar and statistically significant when accounting for pre-exposure trajectory [14]. CSII initiation in Scotland is a diffuse intervention. It is typically preceded by more frequent clinical encounters and attendance at a structured education programme, such as Dose Adjustment For Normal Eating (DAFNE), in the 2 years prior to initiation. In modelling the impact of CSII, we wished to ensure that any within-person trend over calendar time unrelated to CSII could be accounted for in the analysis. In order to evaluate this trend, the HbA_1c_ measurements within the 2 years prior to CSII initiation were excluded since they are related to CSII (see ESM Methods for more details).

Therefore the models estimated the ‘full package’ effect of CSII plus related prior interventions in the 2 year run-up to CSII as they reflect change in HbA_1c_ from before the package started to after it was fully implemented. Models were fitted using R package *nlme* version 3.1–143 [15]. Differential effect of CSII by sociodemographic variable was tested using interaction terms (ESM Methods). Where significant, stratified model results were presented.

Due to the sparse nature of DKA/SHH events, we described the crude event-rate before and after CSII, by dividing the number of events observed by the total number of person-years under observation pre- or post-CSII exposure. To account for repeated occurrence of events within individuals and the background pre-package time-trend, we used generalised linear mixed models with a Poisson likelihood with a random intercept, excluding the 2 years prior to initiation. CSII exposure was included as a binary, time-varying covariate (non-exposed/exposed). All models were adjusted for sex, age and diabetes duration at CSII initiation, baseline HbA_1c_ and pre-package time-trend. Classical likelihood inference for this model relies on approximations of intractable integrals, therefore we used a Bayesian approach [16]. We presented the back-transformed posterior mean (rate ratio) and associated 95% credible intervals (CrI), alongside the posterior probability (pp) of the *β* coefficient associated to CSII exposure being positive or negative in order to quantify the uncertainty around the direction of the effect [17]. Models were implemented using R package *rstan* version 2.19.3 (see ESM Methods for more details).

## Results

### Prevalence

A total of 4941 people ever used CSII between 2004 and the end of 2019.The crude prevalence of ever-CSII users has increased over the past decade from 0.1% in 2004 to 15.1% in 2019. Disparities in CSII usage by the end of 2019 were observed across sociodemographic strata (ESM Fig. 1). Prevalence of ever-CSII users was highest in younger age bands and decreased with age (48.1% in the under 13 age band vs 4.3% in those aged 65 years or older), higher in female (19.9%) vs male participants (11.3%) and twice as high among those living in the least vs the most deprived areas (19.6% in SIMD Q5 vs 10.4% in Q1).

### Baseline cohort characteristics

We analysed glycaemic outcomes in 4684 CSII users who initiated CSII before June 2019. Their baseline characteristics are described in Table 1, alongside those of the non-user sample. Their median overall observation time was 12.4 years; median post-exposure follow-up time was 3.6 years; and 3108 individuals started using flash monitoring/CGM after starting CSII.

**Table 1:** Baseline characteristics of CSII users and matched non-users for analyses of glycaemic outcomes

	CSII users (*N* = 4684)		Matched non-users (*N* = 4141)	
Variable	*n* (%) or median (IQR)	Missingness (%)	*n* (%) or median (IQR)	Missingness (%)
Sex, female	2749 (58.7)	0.0	2438 (58.9)	0.0
Age at initiation	27.5 (12.6, 41.3)	0.0	27.9 (13.5, 41.1)	0.0
Diabetes duration at initiation	11.4 (3.0, 22.9)	0.0	12.0 (3.3, 22.9)	0.0
HbA_1c_ band at baseline		3.8		11.9
< 54 mmol/mol (<7.1%)	529 (11.7)		377 (10.3)	
54–68 mmol/mol (7.1–8.4%)	2117 (47.0)		1264 (34.7)	
69–74 mmol/mol (8.5–8.9%)	713 (15.8)		563 (15.4)	
75–84 mmol/mol (9.0–9.8%)	673 (14.9)		682 (18.7)	
> 84 mmol/mol (>9.8%)	474 (10.5)		761 (20.9)	
*n* HbA_1c_ measurements per individual	20.0 (14.0, 28.0)	0.0	25.0 (15.0, 36.0)	0.0
BMI, kg/m^2^	23.7 (19.4, 27.3)	5.8	23.9 (19.9, 27.9)	14.7
Triacylglycerols, mmol/l	1.0 (0.8, 1.4)	47.0	1.1 (0.8, 1.6)	51.2
Total cholesterol, mmol/l	4.6 (4.0, 5.2)	34.3	4.7 (4.1, 5.3)	40.0
HDL-cholesterol, mmol/l	1.5 (1.3, 1.8)	40.6	1.5 (1.2, 1.8)	46.4
LDL-cholesterol, mmol/l	2.5 (2.0, 3.0)	63.3	2.5 (2.0, 3.0)	67.6
Systolic BP, mmHg	123.5 (114.0, 133.0)	22.0	122.0 (113.0, 132.0)	25.8
Diastolic BP, mmHg	73.0 (67.5, 79.5)	22.0	73.0 (67.0, 79.0)	24.8
CKD-EPI eGFR, ml min^−1^ [1.73 m]^−2^		29.9		35.1
< 15	15 (0.5)		32 (1.2)	
15–30	18 (0.5)		17 (0.6)	
30–60	73 (2.2)		75 (2.8)	
60–90	647 (19.7)		479 (17.8)	
≥ 90	2531 (77.1)		2086 (77.6)	
Albuminuric status: micro/macro albuminuria	618 (21.5)	38.5	541 (25.6)	49.0
SIMD quintile		7.9		8.1
Q1 (most deprived)	618 (14.3)		933 (24.5)	
Q2	787 (18.2)		812 (21.3)	
Q3	933 (21.6)		793 (20.8)	
Q4	944 (21.9)		678 (17.8)	
Q5 (least deprived)	1032 (23.9)		588 (15.5)	
Ever prior DKA admission	1011 (22.0)	1.8	1072 (27.5)	5.7
Ever prior hypoglycaemia admission	497 (10.8)	1.8	387 (9.9)	5.7
Prior FM/CGM usage	389 (8.3)	0.0	–	–

FM, flash monitoring

### HbA1c levels

HbA_1c_ decreased after CSII initiation (ESM Fig.2) from median 66.0 mmol/mol (IQR 58.0, 74.0) (8.2% [IQR 7.5, 8.9]) in the year before initiation to 60.0 mmol/l (54.0, 67.0) (7.6% [7.1, 8.3]) during the first year of CSII exposure and 63.0 (56.0, 71.0) (7.9% [7.3, 8.6]) after ≥5 years. HbA_1c_ levels started decreasing around 2 years prior to CSII initiation, and levels were stable in the preceding years.

Crude within-person differences in HbA_1c_ levels with respect to baseline HbA_1c_ are illustrated in Fig. 1 overall and stratified by age at CSII initiation, sex, SIMD quintile and baseline HbA_1c_. At an individual level, there was a reduction in HbA_1c_ levels post-CSII initiation sustained through time (ESM Table 1): median −5.5 mmol/mol (IQR −12.0, 0.0) (−0.5% [IQR −1.1, 0.0]) (*p* < 0.01) within the first year post-exposure and −5.0 mmol/mol (−12.5, 2.8) (−0.5% [−1.1, 0.3]) (*p* < 0.01) for ≥5 years. Mixed-model results showed no time-trend trajectory of HbA_1c_ prior to CSII-package initiation. CSII initiation was associated with a significant and sustained long-term reduction in HbA_1c_ levels compared with pre-package levels (Table 2). The estimated fold-change was 0.88 overall. For someone with pre-package HbA_1c_ levels of 69 mmol/mol (8.5%), this would correspond to a change of around −8 mmol/mol (−0.7%).

**Figure:**
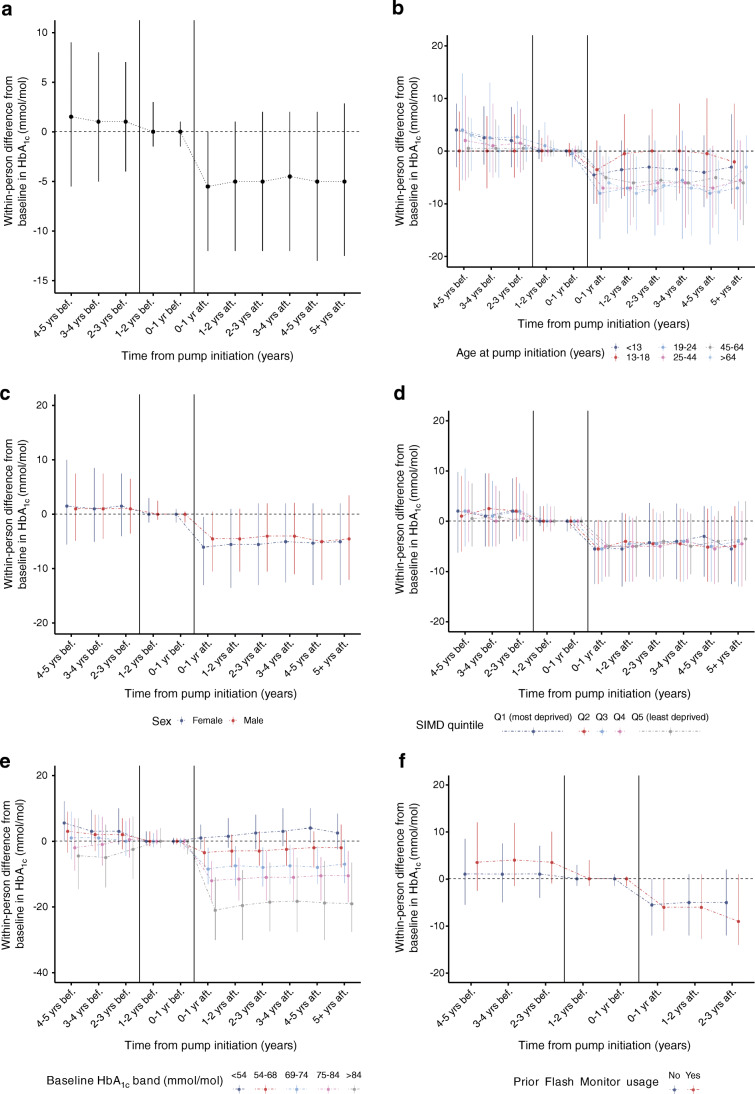

**Table 2:** Modelled estimates of the yearly fold-change in HbA_1c_ in the pre-package time period, and of the fold-change in HbA_1c_ following CSII initiation, compared with pre-package levels, overall and stratified by baseline HbA_1c_ band

Variable	Overall	Baseline HbA_1c_ <54 mmol/mol (<7.1%)	Baseline HbA_1c_ 54–68 mmol/mol (7.1–8.4%)	Baseline HbA_1c_ 69–74 mmol/mol (8.5–8.9%)	Baseline HbA_1c_ 75–84 mmol/mol (9.0–9.8%)	Baseline HbA_1c_> 84 mmol/mol
Time effect, years	1.00 (0.99, 1.00)	0.97 (0.97, 0.98)	0.99 (0.99, 1.00)	1.00 (0.99, 1.00)	1.01 (1.00, 1.02)	1.01 (0.99, 1.02)
CSII exposure time (ref = no CSII usage)						
0–1 year	0.88 (0.87, 0.89)	0.99 (0.95, 1.02)	0.91 (0.89, 0.92)	0.89 (0.86, 0.91)	0.82 (0.79, 0.84)	0.79 (0.76, 0.83)
1–2 years	0.90 (0.89, 0.91)	1.04 (0.99, 1.08)	0.93 (0.91, 0.95)	0.90 (0.87, 0.93)	0.82 (0.79, 0.85)	0.81 (0.76, 0.85)
2–3 years	0.91 (0.90, 0.93)	1.08 (1.02, 1.14)	0.95 (0.93, 0.97)	0.90 (0.87, 0.94)	0.82 (0.79, 0.86)	0.82 (0.76, 0.87)
3–4 years	0.92 (0.91, 0.94)	1.14 (1.08, 1.22)	0.96 (0.93, 0.98)	0.91 (0.87, 0.95)	0.82 (0.78, 0.86)	0.82 (0.76, 0.89)
4–5 years	0.93 (0.91, 0.95)	1.19 (1.11, 1.28)	0.96 (0.93, 0.99)	0.91 (0.86, 0.96)	0.81 (0.77, 0.86)	0.80 (0.73, 0.88)
5 or more years	0.94 (0.92, 0.97)	1.24 (1.14, 1.35)	0.98 (0.95, 1.02)	0.93 (0.88, 0.99)	0.80 (0.75, 0.85)	0.80 (0.72, 0.89)
Number of observations	23,509	2427	10,842	3963	3777	2500
Number of individuals	4099	487	1954	648	595	415

Data are estimates (95% CI)

Pre-package time period refers to 2 years prior to CSII initiation and earlier

Adjusted for sex, age and diabetes duration at CSII initiation, and baseline HbA_1c_

Within-person HbA_1c_ remained stable in the calendar time following pump initiation in the non-user group, with a within-person change of 0.0 mmol/mol (IQR −6.0, 5.5) (0.0% [−0.5, 0.5]) in year 1 to −1.0 mmol/mol (−10.0, 9.0) (−0.1% [−0.9, 0.8]) at ≥5 years (ESM Fig. 3).

CSII exposure effect varied significantly across baseline HbA_1c_, age and sex, and prior flash monitoring/CGM use (*p*_*interaction*_ <0.01 for all), but not across SIMD (*p*_*interaction*_ = 0.25).

Reductions in HbA_1c_ from baseline were observed in all bands ≥54 mmol/mol (7.1%) (Fig. 1, ESM Table 1), whereas HbA_1c_ levels increased slightly in the <54 mmol/mol band. Reductions were significant and sustained through time, with the greatest fall corresponding to the highest baseline HbA_1c_ bands: −21.0 mmol/mol (IQR −30.0, −11.0) (−1.9% [IQR −2.7, −1.0]) (*p* < 0.01) in the first year post-exposure and −19.0 mmol/mol (−27.6, −6.5) (−1.7% [−2.5, −0.6]) at ≥5 years in those with baseline HbA_1c_ >84 mmol/mol (9.8%) (*p* < 0.01). Results from the mixed models were similar (Table 2), with an estimated 0.8-fold-change sustained through time in this group.

Within-person reductions in HbA_1c_ were slightly higher in female than male participants (ESM Tables 2, 3).

Across age, within-person HbA_1c_ reductions were highest among those aged 19–24. Significant reductions were observed in all age bands and sustained through time except in the 13–18 years group. In these adolescents, after an initial decrease, there was no significant difference from baseline beyond the second year. Model estimates showed marginally higher values compared with the expected pre-package HbA_1c_ trajectory (ESM Tables 4, 5). When compared with the matched controls (ESM Fig. 4), HbA_1c_ was lower in the adolescent group in users both before and after CSII initiation. In non-users, HbA_1c_ rose from baseline levels until 3 years post-index: median within-person change from baseline 5.0 mmol/mol (IQR −6.0, 16.5) (0.5% [IQR 0.5, 1.5]) vs −0.0 mmol/mol (−8.4, 8.0) (0.0% [−0.8, 0.7]) in non-users vs users respectively (*p* < 0.01). Levels returned close to baseline in subsequent years.

Within-person reductions in HbA_1c_ did not vary across SIMD (ESM Table 6). They were slightly higher in those with prior flash monitoring/CGM use (ESM Tables 7, 8).

### DKA and SHH events

Overall, 1187 DKA events were observed over 17,479.8 person-years prior to CSII initiation, and 827 DKA events over 16,675.0 person-years after initiation. The crude DKA rate increased prior to package initiation and started decreasing thereafter (ESM Fig. 5, ESM Table 9).

The crude DKA event-rate was significantly lower in CSII-exposed person-time than in non-exposed person-time: 49.6 events (95% CI 46.3, 53.1) per 1000 person-years vs 67.9 (64.1, 71.9).

The Bayesian mixed models confirmed the increasing trend in DKA frequency prior to package initiation and demonstrated that CSII exposure was associated with a reduction in DKA rate compared with the counterfactual: rate ratio 0.61 (95% CrI 0.47, 0.77), with pp of *β* < 0 (i.e. reduction) = 1.00 (ESM Table 10). Hence, we can be very confident that there is a true association.

**Fig. 2:**
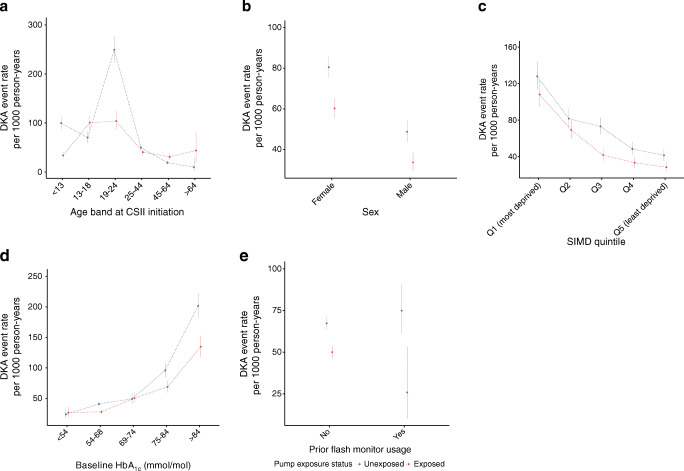
Crude DKA event-rates, by CSII exposure status, stratified by (**a**) age band at CSII initiation, (**b**) sex, (**c**) SIMD quintile, (**d**) baseline HbA_1c_ band, (**e**) prior flash monitoring/CGM usage. Data are crude event rates (95% CI)

Crude DKA rates by CSII exposure status, stratified by baseline HbA_1c_ band, sex, age and SIMD are described in Fig. 2.

Stratified by baseline HbA_1c_ band, significant reductions in DKA rates were observed post-exposure in the 54–68 mmol/mol and ≥ 75 mmol/mol groups (ESM Table 11). Model results were consistent with an association of CSII exposure with a reduction in DKA rate when controlling for pre-intervention trend in the 54–68 mmol/mol and the >84 mmol/mol bands (ESM Table 12).

Crude DKA rates were higher in female than male participants (ESM Table 13) but lower in exposed person-time in both groups. Model results were similar, with CSII being associated with a rate reduction in both groups (ESM Table 14).

Across age band, crude rates were lower in CSII-exposed time, except in the 13–18 years and 45–64 years groups (Fig. 2, ESM Table 15). Model results were generally similar (ESM Table 16). Data were too sparse in the 65 years and older group to draw meaningful conclusions. DKA rates in the 13–18 years group were higher in non-users than users both before and after pump start-date. The crude event-rate in non-users increased from 157.5 (95% CI 139.9, 176.7) pre-index to 244.4 (224.7, 265.4) in the post-index person-time, i.e. as the adolescents get older (ESM Fig. 6). The post-/pre-index increase was similar to that observed in pump users: crude rate ratio 1.55 (95% CI 1.35, 1.79) in non-users vs rate ratio 1.43 (1.16, 1.77) in users.

Higher deprivation levels were associated with increased crude DKA rates. Rates were lower in exposed vs unexposed person-time, though the difference was not always significant (Fig. 2, ESM Table 17). Model results were similar (ESM Table 18).

Crude DKA rates were lower in CSII-exposed time in both those with and without prior flash monitoring/CGM use. Sample size was too low for meaningful inference in the prior flash monitoring/CGM group (ESM Tables 19, 20).

Overall, 451 SHH events were observed in the unexposed person-years, and 296 events in the exposed person-years. The crude SHH rate increased in all years prior to CSII initiation. The crude SHH event-rate was lower in CSII-exposed than non-exposed person-time: 17.8 events per 1000 person-years (95% CI 15.8, 19.9) vs 25.8 (23.5, 28.3). Model estimates from the Bayesian mixed models were consistent with an association of CSII with a reduction in SHH rates compared with pre-package levels: rate ratio 0.67 (95% CrI 0.45, 1.01), pp = 0.97 (ESM Table 21). Hence, we have good confidence that there is a true association. Event numbers were too low to perform stratified analyses.

## Discussion

We investigated the current prevalence of CSII usage among the Scottish population with type 1 diabetes. We studied the association of CSII therapy with HbA_1c_ levels, DKA and SHH event-rates. Prevalence of CSII usage is relatively low, but growing, and varies substantially across sociodemographic strata. CSII therapy was associated with a reduction in HbA_1c_, which was sustained through time for at least 5 years. The greatest reductions were seen in those with the highest baseline HbA_1c_ levels. Effects of exposure did not vary across SIMD quintiles (but CSII prevalence did), while sustained reductions of varying magnitude were seen across sex and age bands, apart from in adolescents. Comparisons with adolescent non-users nonetheless suggested an improvement with CSII in this group. CSII was associated with an overall reduction in DKA rate but the magnitude and direction of effect varied across sociodemographic strata. CSII was associated with an overall reduction in SHH rate.

CSII therapy is generally not funded out-of-pocket in Scotland. There has been a significant increase in CSII provision across the country in the last 8 years in line with a nationwide effort to support the availability of CSII. Variability of usage across sociodemographic strata followed similar patterns to other countries: van den Boom et al. [18] reported higher usage within younger age bands in the Diabetes Patienten Verlaufsdokumentation (DPV) cohort. The National Diabetes Insulin Pump Audit [19] (England, Wales) 2017–2018 reported a higher pump-use prevalence in women and in those from less deprived areas. Findings from a qualitative study by Scott et al. [20] highlighting higher access barriers to intensive insulin regimen (including CSII therapy) for people from lower socioeconomic groups are reflected in our observation that CSII prevalence is lower among the most deprived.

Within-person changes observed in our national study following CSII initiation were comparable in direction and magnitude to those reported regionally in NHS Lothian [7]. We found the CSII therapy package to be independently associated with a significant fall in HbA_1c_. Magnitude and direction of estimated changes are similar to those reported in before/after studies of patients switching from multiple daily injections (MDI) to CSII in Pickup and Sutton’s meta-analysis [1]: −0.72% (−7.9 mmol/mol). Our findings are in line with those from other local-scale UK studies which reported improvements in glycaemic control following CSII initiation to be sustained at least up to 5 to 6 years [7, 21, 22]. While diabetes care in Scotland has improved in the past decade, the stability of HbA_1c_ in the matched non-user sample supports conclusions that observed reductions among pump users are associated with CSII-package and not explained by the secular trend of HbA_1c_ improvement.

In line with previous findings, those with the highest baseline HbA_1c_ benefited most from CSII. NHS Lothian reported a −22.2 mmol/mol change in those starting with HbA_1c_ ≥ 85 mmol/mol and the meta-analysis showed a mean difference (MDI vs CSII) greater than 16 mmol/mol for those with baseline HbA_1c_ ≥ 80 mmol/mol [1, 23]. Hence, CSII helps individuals transition from higher to lower complication-risk HbA_1c_ groups. The slight increase in HbA_1c_ in those with baseline levels <54 mmol/mol is unsurprising as it is likely that this group would have been prescribed CSII to reduce hypoglycaemia.

Differences observed across sex strata were similar to previous findings [7, 24].

Patterns in HbA_1c_ among those starting a pump in their teenage years were similar to those described by Johnson et al. in their long-term paediatric CSII outcome study (median age 11.5 years) [2]. In their study, after an initial decrease, HbA_1c_ started increasing but remained lower than levels of matched control participants on MDI, and differences remained significant for 7 years. Our study’s matched differences stopped being significant after 4 years, likely due to our group including older individuals, who, after adolescence, transition into a phase of more stable glycaemic control, whereas participants in the study by Johnson et al. were censored when they transitioned to adult services. Related to this, the higher reductions among the 19–24 years age band are likely due to a combination of glycaemic control tending to stabilise at lower levels beyond adolescence, and CSII use.

Although the sample size was small, we found that CSII was beneficial in those initiating CSII after prior flash monitoring/CGM usage.

We found CSII to be associated with a reduced overall DKA rate, whereas previous findings have been conflicting. A meta-analysis [25] suggested that CSII was associated with higher DKA rates in older studies (before 1993). Pickup and Keen [26] noted that modifiable factors such as patient/healthcare provider inexperience can increase the likelihood of DKA around the beginning of CSII use, as ketosis occurs rapidly following insulin interruption due to use of rapid-acting insulin in CSII devices [4], but we found no increase of crude DKA rates within the first year of usage.

More recent studies have been inconclusive. Some paediatric studies reported decreases, and others reported increases [2–4, 27]. Thomas et al. [28] identified CSII as a predictor of DKA in adults from the FinnDiane cohort. Different follow-up times, DKA definitions (e.g. pH/bicarbonate-based) and cohort characteristics (adults/children) could partially explain the differing findings between studies. This variability is reflected within our study by the difference in the direction of estimates across age bands, with rate increase in the 45–64 years age band, for example.

The increase in risk of DKA with deprivation levels in Scotland was highlighted by Govan et al. [29]. Shulman et al. [30] reported worse outcomes in those from lower socioeconomic status in a Canadian paediatric study of pump users. However, our findings show that although DKA rates remain higher in more vs less deprived areas in post-pump person-time, they were generally lower in post- vs pre-pump person-time. Where differences were not significant, rates did not increase post-pump.

Since recurrent DKA is an exclusion criterion for pump therapy in Scotland, our conclusions pertain to a group of people with type 1 diabetes who have a lower pre-exposure propensity to DKA and may not generalise to those with a higher baseline DKA propensity. It is crucial to understand the mechanisms behind the observed reduction in DKA.

Several studies [1–3] have shown an association of CSII with a reduction in severe hypoglycaemia rate (a 4.34 rate-reduction factor was reported by Pickup and Sutton [1]). Crude SHH rates were lower in CSII-exposed vs non-exposed person-time, and the modelled estimate was consistent with an improvement in SHH following CSII-package. However, our findings are not comparable to other studies, which captured severe hypoglycaemia events requiring third-party assistance, whereas our definition was limited to hospital admissions, missing any events treated in the community.

### Strengths

This is one of the largest real-world, contemporary, nationwide assessments of the prevalence of CSII and its effect on glycaemic outcomes, combining near-complete capture of data on people with type 1 diabetes nationally, long follow-up time and extensive analyses.

### Limitations

The main limitations of our study are the lack of data in older people, incomplete capture of self-funded flash monitoring use and non-capture of non-hospitalised severe hypoglycaemia [31]. CSIIs can be prescribed for debilitating hypoglycaemia in adults or children older than 12, as recommended by NICE [6], but we could not evaluate hypoglycaemia as an effectiveness outcome for CSII therapy.

In Scotland, eligibility for receiving CSII on the NHS has evolved over time: from 2011 there was a major push to widen provision, and the clinical and sociodemographic profile of those receiving pumps has changed. However, the vast majority of CSII initiation occurred since 2011, with most of our data reflecting those initiating pump therapy in the period 2013–2017 and followed up to the present day (median start date 5 December 2014 [IQR 16 June 2013, 27 February 2017]). Therefore, our results reflect the contemporary profile and outcomes of those in receipt of CSII therapy which is most relevant to current effectiveness and safety estimation.

The majority of CSII users had their person-time censored for start of flash monitoring/CGM. The person-time lost to follow-up was relatively low and unlikely to affect the analyses since the step-change in HbA_1c_ occurs within the first year post-CSII initiation.

Generalisability of findings to all people with type 1 diabetes is limited by the allocation bias resulting from the selection process for pump therapy.

Our HbA_1c_ analyses are susceptible to regression to the mean. However, the magnitude of pre–post change among never-users indicates that only a small proportion of the decrease observed in users following CSII initiation would actually be attributable to this artefact. Finally, as with all observational analyses, our estimates may be affected by unmeasured confounding and measurement error, although the self-controlled nature of some analyses eliminates time-invariant confounding.

### Conclusions

Our study shows that CSII therapy is associated with improvements in HbA_1c_, DKA and SHH among pump users with type 1 diabetes in Scotland. The improvements associated with CSII therapy suggest that a more widespread use of pump therapy has the potential to reduce HbA_1c_ levels and DKA rates further. Our study highlights the importance of CSII technology reaching those with suboptimal glycaemic control and those from most deprived areas. This latter group, with historically poorer glycaemic control [10], stands to benefit from CSII usage, as we have not found evidence of CSII effects being worse in people from more deprived areas (in contrast to their CSII usage rates). Future research is needed to assess the effect of SIMD on outcomes in specific subgroups. Generalisation of our findings to all people with type 1 diabetes in Scotland is limited by the specific eligibility criteria to CSII. Hence, it is crucial to identify the determinants of good response to CSII to optimise a targeted wider roll-out. Moreover, other outcomes that we cannot directly quantify need to be considered, such as improvement in quality of life and reduction in glucose variability. Motivation is critical to successful CSII usage [23], and it would be interesting to examine how outcomes differ in a known-to-be-motivated group (pregnant women). Finally, future research is warranted to examine how the rapid evolution of technology (integrated pump-and-CGM systems with alarms and/or suspension systems, hybrid/DIY closed loops) affects DKA and severe hypoglycaemia rates.

## Supplementary Information

ESM (PDF 889 kb)

## Data Availability

We do not have governance permissions to share individual-level data on which these analyses were conducted. However, for any genuine requests to audit the validity of the analyses, the verifiable research pipeline that we operate allows researchers to make a request to the corresponding author to view the analyses being run and the resulting tabulations, summary statistics and parameter estimates.

## Abbreviations

CGM: Continuous glucose monitoring
CrI: Credible interval
CSII: Continuous subcutaneous insulin infusion
DKA: Diabetic ketoacidosis
MDI: Multiple daily injections
NHS: National Health Service
NICE: National Institute for Health and Care Excellence
pp: Posterior probability
SCI-Diabetes: Scottish Care Information-Diabetes
SHH: Severe hospitalised hypoglycaemia
SIMD: Scottish Index of Multiple Deprivation
