# Supplementary material for: Marked improvements in glycaemic outcomes following insulin pump therapy initiation in people with type 1 diabetes: a nationwide observational study in Scotland
Source: Diabetologia. 2021 Mar 8;64(6):1320–31. doi: 10.1007/s00125-021-05413-7 (PMC8099793; doi:10.1007/s00125-021-05413-7)
Supplement: Supplementary file 1 — (PDF 889 kb) [file 125_2021_5413_MOESM1_ESM.pdf]

Supplementary Material for Marked improvements in glycaemic outcomes following insulin pump therapy initiation in people with Type 1 Diabetes - a nationwide observational study in Scotland

A. Jeyam F.W. Gibb J.A. McKnight B. Kennon J.E. O'Reilly T.M. Caparrotta A. Höhn S.J. McGurnaghan L.A.K. Blackbourn S. Hatam R.J. McKrimmon G. Leese S. Philip R.S. Lindsay J. Chalmers J. Petrie S.H. Wild N. Sattar P. M. McKeigue H. M. Colhoun On behalf of the SDRN Epi Group

Methods

Study population

Individuals were censored for the following reasons: end of study (26.6%), end of observability (3.8%), death (0.9%), start of FM/CGM (62.0%), stopping CSII (6.7%).

Exposure, outcomes and covariates

Diabetic ketoacidosis (DKA) events were defined as any hospital admission or death, involving an ICD10 diagnosis code of E10.1, E11.1, E12.1, E13.1 or E14.1 at any level of the reasons for admission or causes of death.

Severe hypoglycaemic (SHH) events were defined as any hospital admission or death involving an ICD10 diagnosis code of E15, E16.0, E16.1, E16.2 at any level of the reasons for admission or causes of death.

Statistical Analysis

We modelled the log-transformed HbA_{1c}, with fixed effects defined as age at CSII initiation, diabetes duration at CSII initiation, sex, log(baseline HbA_{1c}), time (in years and centered on CSII initiation such that CSII starts at time=0) and categorical CSII-exposure time (years, with 0=non-exposed as the reference category); the CSII-exposure time effects estimated by the model are the estimated differences in log(HbA_{1c}) at the various timepoints, compared to the levels individuals would have had if the within-person pre-intervention trend occurring in HbA_{1c} had continued post-exposure. The within-individual error-correlation structure was specified as a continuous first-order autoregressive (CAR1) structure to allow for exponentially decayed correlation in measurements the further apart they are in time.

We formally tested the significance of interaction terms using fully adjusted versions of the mixed models described previously. Fixed effects included were: sex, SIMD, diabetes duration, baseline HbA_{1c}, categorical CSII-time and the interaction term (categorical CSII-time)*group of interest. Significance of the interaction term was assessed using Likelihood Ratio Tests (LRT) and models were fitted using Maximum Likelihood (ML) for this purpose so as to appropriately compare models with different fixed effects.

In order to crudely evaluate overall trends that might occur in HbA_{1c} during similar calendar time in non-users and more specifically, the worsening glycaemic control with age among the adolescent subgroup, we used a 1:1 matched non-user sample as control group. For each CSII user from the population under study, we randomly selected an individual matched in age, sex and diabetes duration, who was not using any device (CSII/FM/CGM)

at the CSII user's CSII start date and for at least one year thereafter, and assigning the user's CSII start date as their index date. We described HbA_{1c} levels over time in users and non-users, and compared paired levels and differences from baseline in post-index years (i.e. changes as the individuals get older) between users and matched non-users using one sided Wilcoxon signed-rank test adjusted for multiple comparisons.

For DKA, we described crude event-rates in both pre and post-index time for both groups and compared the post vs pre rate ratio in users and non-users.

For the Bayesian Poisson mixed-models, we used a QR reparametrisation for the fixed effects. The reparametrised parameters were given Gaussian priors (mean=0,sd=100). Individual random effects were modelled as standard normal variates shifted by an intercept and scaled by a standard deviation. The prior chosen for the standard deviation of the individual random effects was a half-Cauchy(0,5).

Results

ESM Table 1: Within-person change from baseline in HbA_{1c} (mmol/mol) over time from CSII initiation (years) - overall and stratified by baseline HbA_{1c} band - N and median (IQR)

Time from pump initiation (yrs)	Overall		Baseline HbA1c <54 mmol/mol[7.1%]		Baseline HbA1c 54-68 mmol/mol[8.4%]		Baseline HbA1c 69[8.5%]-74 mmol/mol [8.9%]		Baseline HbA1c 75[9.0%]-84 mmol/mol [9.8%]		Baseline HbA1c >84 mmol/mol	
	N	Med. (IQR)	N	Med. (IQR)	N	Med. (IQR)	N	Med. (IQR)	N	Med. (IQR)	N	Med. (IQR)
4-5 yrs bef.	2953	1.5 (-5.5,9.0)	264	5.5 (0.0,12.2)	1278	3.0 (-3.5,9.0)	516	1.0 (-5.5,9.0)	500	-2.0 (-9.0,7.0)	368	-4.5 (-14.5,7.0)
3-4 yrs bef.	3218	1.0 (-5.0,8.0)	298	3.0 (-1.0,9.5)	1400	2.0 (-3.0,8.0)	559	1.0 (-5.0,8.0)	544	-1.0 (-7.5,7.5)	385	-5.0 (-14.0,5.0)
2-3 yrs bef.	3547	1.0 (-4.0,7.0)	348	3.0 (-1.0,10.0)	1580	2.0 (-2.5,7.0)	603	0.0 (-4.5,6.1)	574	0.5 (-5.0,7.0)	404	-2.5 (-11.5,7.5)
1-2 yrs bef.	4081	ref.	459	ref.	1895	ref.	661	ref.	613	ref.	418	ref.
0-1 yr bef.	4592	ref.	529	ref.	2116	ref.	713	ref.	673	ref.	473	ref.
0-1 yr aft.	4602	-5.5 (-12.0,0.0)**	528	1.0 (-2.5,5.0)	2103	-3.5 (-8.0,1.0)**	711	-8.5 (-13.0,-2.0)**	672	-12.0 (-18.0,-6.0)**	468	-21.0 (-30.0,-11.0)**
1-2 yrs aft.	3904	-5.0 (-12.0,1.0)**	425	1.5 (-2.0,7.0)	1760	-3.0 (-8.0,2.0)**	609	-7.5 (-13.5,-1.5)**	591	-11.5 (-18.0,-5.0)**	395	-19.5 (-30.0,-8.8)**
2-3 yrs aft.	3198	-5.0 (-12.0,2.0)**	317	2.5 (-2.5,8.0)	1395	-3.0 (-7.5,3.0)**	521	-8.0 (-13.8,-1.9)**	504	-11.0 (-18.0,-3.0)**	336	-18.5 (-27.5,-6.5)**
3-4 yrs aft.	2668	-4.5 (-12.0,2.0)**	242	3.0 (-1.5,10.0)	1142	-2.5 (-7.5,3.0)**	441	-7.5 (-13.0,-1.0)**	431	-11.0 (-18.0,-2.0)**	293	-18.2 (-27.6,-5.0)**
4-5 yrs aft.	2068	-5.0 (-13.0,2.0)**	169	4.0 (-0.5,10.0)	850	-2.0 (-8.0,3.0)**	356	-8.0 (-13.0,-1.5)**	345	-10.5 (-18.0,-4.9)**	236	-18.8 (-30.0,-6.0)**
5+ yrs aft.	1392	-5.0 (-12.5,2.8)**	120	2.5 (-2.5,8.4)	572	-2.0 (-8.0,5.0)**	237	-7.0 (-12.8,0.5)**	220	-10.5 (-18.5,-3.0)**	151	-19.0 (-27.6,-6.5)**

Note:

For post-exposure years significance of one-sided Wilcoxon signed-rank test p adjusted for multiple comparisons is denoted by ** ($p<0.01$) or * ($0.01 \leq p<0.05$)

ESM Table 2: Within-person change from baseline in HbA_{1c} (mmol/mol) over time from CSII initiation (years), stratified by sex - N and median (IQR) - For post-exposure years significance of one-sided Wilcoxon signed-rank test p adjusted for multiple comparisons is denoted by ** ($p<0.01$) or * ($0.01 \leq p<0.05$)

Time from CSII initiation (yrs)	Female		Male	
	N	Med. (IQR)	N	Med. (IQR)
4-5 yrs bef.	1811	1.5 (-5.5,10.0)	1142	1.0 (-4.9,7.5)
3-4 yrs bef.	1968	1.0 (-5.0,8.5)	1250	1.0 (-4.5,7.5)
2-3 yrs bef.	2139	1.5 (-4.0,7.5)	1408	1.0 (-3.5,6.5)
1-2 yrs bef.	2409	ref.	1672	ref.
0-1 yr bef.	2695	ref.	1897	ref.
0-1 yr aft.	2702	-6.0 (-13.0,-0.5)**	1900	-4.5 (-10.5,0.5)**
1-2 yrs aft.	2296	-5.5 (-13.5,1.0)**	1608	-4.5 (-10.5,1.0)**
2-3 yrs aft.	1901	-5.5 (-13.0,2.0)**	1297	-4.0 (-10.5,2.0)**
3-4 yrs aft.	1603	-5.0 (-12.5,2.0)**	1065	-4.0 (-11.0,2.1)**
4-5 yrs aft.	1251	-5.2 (-13.0,2.0)**	817	-5.0 (-12.0,1.0)**
5+ yrs aft.	829	-5.0 (-13.0,2.0)**	563	-4.5 (-12.0,3.5)**

ESM Table 3: Modelled estimates (95% CI) of the yearly fold-change in HbA_{1c} in the pre-package time-period (2 years prior to CSII initiation and earlier), and of the fold-change in HbA_{1c} following CSII initiation, compared to pre-package levels - overall and stratified by sex - adjusting for age, diabetes duration at CSII initiation and HbA_{1c} at baseline -

Variable	Female	Male
Time effect (years)	1.00 (0.99; 1.00)	0.99 (0.99; 1.00)
CSII exposure time		
ref= No CSII usage		
0-1 year	0.87 (0.86; 0.88)	0.91 (0.89; 0.92)
1-2 years	0.89 (0.87; 0.90)	0.92 (0.90; 0.94)
2-3 years	0.90 (0.88; 0.92)	0.94 (0.91; 0.96)
3-4 years	0.91 (0.89; 0.94)	0.95 (0.92; 0.97)
4-5 years	0.92 (0.89; 0.94)	0.95 (0.91; 0.98)
5 or more years	0.93 (0.89; 0.96)	0.96 (0.93; 1.00)
Number of observations	14022	9487
Number of individuals	2386	1713

ESM Table 4: Within-person change from baseline in HbA_{1c} (mmol/mol) over time from CSII initiation (years), stratified by age band at CSII initiation - N and median (IQR)

Time from pump initiation (yrs)	Age <13 y.o.		Age 13-18 y.o.		Age 19-24 y.o.		Age 25-44 y.o.		Age 45-64 y.o.		Age >64 y.o.	
	N	Med. (IQR)	N	Med. (IQR)	N	Med. (IQR)	N	Med. (IQR)	N	Med. (IQR)	N	Med. (IQR)
4-5 yrs bef.	212	4.0 (-3.0,9.0)	302	0.0 (-7.5,7.5)	248	4.0 (-5.5,14.8)	1401	2.0 (-5.6,10.5)	733	0.5 (-5.0,6.5)	57	3.0 (-3.8,6.2)
3-4 yrs bef.	314	2.5 (-2.5,8.5)	352	0.0 (-7.0,6.6)	267	2.5 (-4.0,13.0)	1463	1.0 (-5.0,9.0)	764	0.5 (-4.0,6.0)	58	0.0 (-5.0,4.9)
2-3 yrs bef.	487	2.0 (-3.0,8.4)	420	0.0 (-5.0,7.0)	281	2.7 (-3.0,9.5)	1516	1.5 (-4.0,8.0)	784	0.5 (-3.5,5.0)	59	0.5 (-2.0,4.8)
1-2 yrs bef.	862	ref.	500	ref.	303	ref.	1553	ref.	802	ref.	61	ref.
0-1 yr bef.	1203	ref.	552	ref.	321	ref.	1624	ref.	828	ref.	64	ref.
0-1 yr aft.	1205	-4.5 (-10.0,0.0)**	559	-3.5 (-10.0,2.0)**	323	-8.0 (-16.6,1.0)**	1624	-7.0 (-13.5,-1.0)**	827	-5.0 (-11.5,0.0)**	64	-6.0 (-10.8,-1.0)**
1-2 yrs aft.	980	-3.5 (-9.0,2.0)**	506	-0.5 (-8.5,7.0)	274	-7.0 (-15.6,0.5)**	1397	-7.0 (-14.0,-0.5)**	698	-6.0 (-12.5,-0.5)**	49	-8.0 (-15.0,-1.0)**
2-3 yrs aft.	756	-3.0 (-10.0,3.0)**	437	0.0 (-8.4,8.0)	227	-7.5 (-16.0,2.0)**	1162	-6.0 (-14.0,0.0)**	579	-5.5 (-11.5,-0.6)**	37	-6.5 (-14.0,-1.5)**
3-4 yrs aft.	614	-3.4 (-9.4,3.0)**	380	0.0 (-8.0,9.0)	185	-5.5 (-16.6,3.9)**	962	-6.0 (-14.5,1.0)**	495	-6.0 (-12.0,-1.0)**	32	-7.0 (-16.0,0.5)*
4-5 yrs aft.	458	-4.0 (-10.6,3.0)**	305	-0.5 (-9.0,10.0)	137	-8.0 (-17.8,0.5)**	741	-7.0 (-14.5,1.0)**	404	-5.0 (-12.0,-0.5)**	23	-7.8 (-15.8,-1.8)
5+ yrs aft.	305	-3.0 (-10.0,7.0)**	218	-2.0 (-11.0,9.0)	91	-7.0 (-17.0,2.1)**	498	-5.5 (-13.0,2.0)**	268	-6.0 (-14.0,0.0)**	12	-3.0 (-10.0,3.0)

Note:

For post-exposure years, significance of one-sided Wilcoxon signed-rank test p adjusted for multiple comparisons is denoted by ** ($p<0.01$) or * ($0.01 \leq p<0.05$)

ESM Table 5: Modelled estimates (95% CI) of the yearly fold-change in HbA_{1c} in the pre-package time-period (2 years prior to CSII initiation and earlier), and of the fold-change in HbA_{1c} following CSII initiation compared to the pre-package years -stratified by age band at pump initiation, adjusting by age, sex, diabetes duration and HbA_{1c} at baseline

Variable	<13 y.o.	13-18 y.o.	19-24 y.o.	25-44 y.o.	45-64 y.o.	>64 y.o.
Time effect (years)	0.99 (0.98; 0.99)	1.00 (0.99; 1.01)	1.00 (0.99; 1.01)	1.00 (0.99; 1.00)	1.00 (0.99; 1.00)	1.00 (0.99; 1.02)
CSII exposure time						
ref= No CSII usage						
0-1 year	0.90 (0.88; 0.93)	0.93 (0.89; 0.96)	0.83 (0.79; 0.87)	0.87 (0.85; 0.89)	0.90 (0.88; 0.92)	0.89 (0.84; 0.94)
1-2 years	0.94 (0.91; 0.97)	0.98 (0.93; 1.02)	0.85 (0.80; 0.91)	0.87 (0.85; 0.89)	0.90 (0.87; 0.92)	0.88 (0.81; 0.95)
2-3 years	0.96 (0.92; 1.00)	1.00 (0.95; 1.06)	0.86 (0.80; 0.93)	0.88 (0.86; 0.91)	0.90 (0.88; 0.93)	0.87 (0.79; 0.95)
3-4 years	0.98 (0.93; 1.03)	1.03 (0.96; 1.10)	0.87 (0.79; 0.95)	0.90 (0.87; 0.92)	0.90 (0.87; 0.93)	0.86 (0.78; 0.96)
4-5 years	1.00 (0.94; 1.06)	1.03 (0.95; 1.11)	0.85 (0.76; 0.94)	0.90 (0.86; 0.93)	0.91 (0.87; 0.94)	0.84 (0.74; 0.95)
5 or more years	1.05 (0.98; 1.12)	1.05 (0.96; 1.15)	0.83 (0.74; 0.94)	0.91 (0.87; 0.94)	0.90 (0.86; 0.95)	0.83 (0.72; 0.96)
Number of observations	4826	2947	1540	8867	4966	363
Number of individuals	1100	512	275	1413	742	57

ESM Table 6: Within-person change from baseline in HbA_{1c} (mmol/mol), over time from CSII initiation (years), stratified by SIMD quintile - N and median (IQR)

Time from pump initiation (yrs)	SIMD Q1 (most deprived)		Q2		Q3		Q4		Q5 (least deprived)	
	N	Med. (IQR)	N	Med. (IQR)	N	Med. (IQR)	N	Med. (IQR)	N	Med. (IQR)
4-5 yrs bef.	376	2.0 (-6.2,9.8)	497	1.0 (-6.0,9.0)	558	2.0 (-5.0,10.5)	559	2.0 (-4.1,8.0)	641	0.5 (-5.0,7.5)
3-4 yrs bef.	407	1.0 (-5.0,9.5)	551	2.5 (-5.0,9.5)	630	1.0 (-5.0,8.0)	608	0.0 (-5.0,7.0)	694	0.8 (-4.5,6.0)
2-3 yrs bef.	443	2.0 (-4.0,8.5)	619	2.0 (-3.5,8.8)	689	2.0 (-3.0,7.5)	686	0.5 (-4.0,6.0)	769	0.0 (-4.0,5.5)
1-2 yrs bef.	527	ref.	700	ref.	809	ref.	803	ref.	886	ref.
0-1 yr bef.	604	ref.	774	ref.	906	ref.	930	ref.	1010	ref.
0-1 yr aft.	605	-5.5 (-12.5,0.0)**	773	-5.5 (-12.5,0.2)**	911	-5.5 (-12.0,0.0)**	934	-5.0 (-11.0,0.0)**	1014	-5.0 (-11.0,0.0)**
1-2 yrs aft.	494	-5.5 (-13.0,1.7)**	645	-4.0 (-12.0,1.5)**	769	-4.5 (-12.0,1.0)**	808	-5.0 (-12.0,0.6)**	871	-5.0 (-11.0,1.0)**
2-3 yrs aft.	396	-4.2 (-11.0,3.6)**	512	-4.5 (-11.5,2.5)**	623	-4.5 (-12.0,1.8)**	660	-5.0 (-11.5,1.5)**	723	-4.0 (-11.0,2.0)**
3-4 yrs aft.	340	-4.0 (-11.5,3.5)**	429	-4.5 (-12.0,2.5)**	518	-4.0 (-12.0,2.5)**	534	-4.0 (-11.5,1.9)**	600	-5.0 (-10.5,1.5)**
4-5 yrs aft.	271	-3.0 (-11.0,3.0)**	339	-5.2 (-12.0,2.5)**	409	-5.0 (-12.0,3.0)**	404	-5.5 (-12.5,1.0)**	442	-4.0 (-12.1,2.0)**
5+ yrs aft.	177	-5.5 (-12.5,1.0)**	211	-5.0 (-12.0,3.0)**	264	-4.0 (-13.0,4.0)**	286	-4.5 (-13.0,2.0)**	312	-3.5 (-11.8,4.0)**

Note:

For post-exposure years, significance of one-sided Wilcoxon signed-rank test p adjusted for multiple comparisons is denoted by ** ($p<0.01$) or * ($0.01 \leq p<0.05$)

ESM Table 7: Within-person change from baseline in HbA_{1c} (mmol/mol), over time from CSII initiation (years), stratified by prior FM/CGM usage - N and median (IQR)

Time from pump initiation (yrs)	No prior FM/CGM use		Prior FM/CGM use	
	N	Med. (IQR)	N	Med. (IQR)
4-5 yrs bef.	2723	1.0 (-5.5,8.5)	230	3.5 (-2.5,12.0)
3-4 yrs bef.	2967	1.0 (-5.0,7.5)	251	4.0 (-1.5,11.9)
2-3 yrs bef.	3266	1.0 (-4.0,7.0)	281	3.5 (-1.0,10.0)
1-2 yrs bef.	3742	ref.	339	ref.
0-1 yr bef.	4203	ref.	389	ref.
0-1 yr aft.	4213	-5.5 (-12.0,0.0)**	389	-6.0 (-11.0,0.0)**
1-2 yrs aft.	3684	-5.0 (-12.0,1.0)**	220	-6.0 (-12.8,1.0)**
2-3 yrs aft.	3144	-5.0 (-12.0,2.0)**	54	-9.0 (-14.0,1.0)**
3-4 yrs aft.	2653	-4.5 (-12.0,2.0)**	15	-6.0 (-18.1,-4.5)**
4-5 yrs aft.	2065	-5.0 (-13.0,2.0)**	<10	-
5+ yrs aft.	1390	-5.0 (-12.5,2.8)**	<10	-

Note:

For post-exposure years, significance of one-sided Wilcoxon signed-rank test p adjusted for multiple comparisons is denoted by ** ($p<0.01$) or * ($0.01 \leq p<0.05$)

ESM Table 8: Modelled estimates (95% CI) of the yearly fold-change in HbA_{1c} in the pre-package time-period (2 years prior to CSII initiation and earlier), and of the fold-change in HbA_{1c} following CSII initiation, compared to pre-package levels - overall and stratified by prior FM/CGM use - adjusting for age, sex, diabetes duration and HbA_{1c} at baseline -

Variable	No prior FM/CGM use	Prior FM/CGM use
Time effect (years)	1.00 (0.99; 1.00)	0.99 (0.98; 1.00)
CSII exposure time		
ref= No CSII usage		
0-1 year	0.89 (0.88; 0.90)	0.85 (0.81; 0.89)
1-2 years	0.90 (0.89; 0.92)	0.86 (0.81; 0.91)
2-3 years	0.92 (0.90; 0.93)	0.87 (0.80; 0.94)
3-4 years	0.93 (0.91; 0.95)	0.88 (0.79; 0.99)
4-5 years	0.93 (0.91; 0.95)	0.95 (0.77; 1.19)
5 or more years	0.94 (0.92; 0.97)	0.99 (0.78; 1.27)
Number of observations	22273	1236
Number of individuals	3728	371

ESM Table 9: DKA and SHH - Crude event-rates and associated 95% Confidence Intervals, by time from CSII initiation (years) - DKA and SHH - (E denotes the number of events observed and PY the number of person-years observed)

Time from CSII initiation (years)	SHH			DKA		
	E	PY	Rate (95% CI)	E	PY	Rate (95% CI)
4-5 yrs bef.	55	2853.9	19.3 (14.5 ; 25.1)	177	2853.9	62.0 (53.2 ; 71.9)
3-4 yrs bef.	68	3086.4	22.0 (17.1 ; 27.9)	204	3086.4	66.1 (57.3 ; 75.8)
2-3 yrs bef.	76	3372.7	22.5 (17.8 ; 28.2)	256	3372.7	75.9 (66.9 ; 85.8)
1-2 yrs bef.	108	3804.2	28.4 (23.3 ; 34.3)	270	3804.2	71.0 (62.8 ; 80.0)
0-1 yr bef.	144	4365.0	33.0 (27.8 ; 38.8)	280	4365.0	64.1 (56.9 ; 72.1)
0-1 yr aft.	87	4089.0	21.3 (17.0 ; 26.2)	221	4089.0	54.0 (47.2 ; 61.7)
1-2 yrs aft.	56	3370.8	16.6 (12.5 ; 21.6)	175	3370.8	51.9 (44.5 ; 60.2)
2-3 yrs aft.	54	2834.7	19.0 (14.3 ; 24.9)	117	2834.7	41.3 (34.1 ; 49.5)
3-4 yrs aft.	37	2292.5	16.1 (11.4 ; 22.2)	132	2292.5	57.6 (48.2 ; 68.3)
4-5 yrs aft.	20	1607.1	12.4 (7.6 ; 19.2)	80	1607.1	49.8 (39.5 ; 62.0)
5+ yrs aft.	42	2483.3	16.9 (12.2 ; 22.9)	102	2483.3	41.1 (33.5 ; 49.9)

ESM Table 10: Estimated Rate Ratios of DKA admissions for CSII-exposed person-time vs pre-package person-time and for pre-package trend from models adjusted for age, sex, diabetes duration and baseline HbA1c - Posterior mean (95% Credible Interval) and posterior probability of the direction of the effect (pp)

Variable	Rate Ratios - Posterior mean (95% Credible Interval)	pp
Time effect (years)	1.03 (0.99 ; 1.07)	0.95
Pump-exposure status		
ref= Unexposed		
Exposed	0.61 (0.47 ; 0.77)	1.00

ESM Table 11: Crude DKA event-rates and associated 95% Confidence Intervals by CSII exposure status - Stratified by baseline HbA$_{1c}$ bands - (E denotes the number of events observed and PY the number of person-years observed) -

CSII Exposure status	E|PY| Crude event rate (95% CI)				
	<54 mmol/mol[7.1%]	54-68[8.4%] mmol/mol	69[8.5%]-74[8.9%] mmol/mol	75[9.0%]-84[9.8%] mmol/mol	>84 mmol/mol
No exposure	42 | 1749.7 | 24.0 (17.3 ; 32.4)	320 | 7825.7 | 40.9 (36.5 ; 45.6)	145 | 2942.6 | 49.3 (41.6 ; 58.0)	269 | 2800.9 | 96.0 (84.9 ; 108.2)	400 | 1985.0 | 201.5 (182.2 ; 222.3)
Exposed	43 | 1601.5 | 26.8 (19.4 ; 36.2)	200 | 7143.1 | 28.0 (24.3 ; 32.2)	137 | 2706.7 | 50.6 (42.5 ; 59.8)	185 | 2677.7 | 69.1 (59.5 ; 79.8)	239 | 1777.3 | 134.5 (118.0 ; 152.6)

ESM Table 12: Estimated Rate Ratios of DKA admissions for CSII-exposed person-time vs pre-package person-time and for pre-package trend from models adjusted for age, sex and diabetes duration - stratified by baseline HbA_{1c} band - Posterior mean (95% Credible Interval) and posterior probability of the direction of the effect (pp)

CSII Exposure status	<54 mmol/mol [7.1\%]		54-68 mmol/mol [8.4\%]		69[8.5\%]-74[8.9\%] mmol/mol		75[9.0\%]-84[9.8\%] mmol/mol		>84 mmol/mol	
	PM (95% CrI)	pp	PM (95% CrI)	pp	PM (95% CrI)	pp	PM (95% CrI)	pp	PM (95% CrI)	pp
Time effect (years)	1.27 (1.09 ; 1.47)	1.00	1.03 (0.95 ; 1.10)	0.78	1.03 (0.94 ; 1.13)	0.76	0.94 (0.86 ; 1.02)	0.93	1.06 (0.99 ; 1.14)	0.95
Pump-exposure status										
ref= Unexposed										
Exposed	0.23 (0.07 ; 0.74)	0.99	0.51 (0.32 ; 0.83)	1.00	0.73 (0.38 ; 1.35)	0.85	1.16 (0.65 ; 2.01)	0.69	0.51 (0.32 ; 0.80)	1.00

ESM Table 13: Crude DKA event-rates and associated 95% Confidence Intervals by CSII exposure status - Stratified by sex - (E denotes the number of events observed and PY the number of person-years observed) -

Pump Exposure status	E|PY| Crude event rate (95% CI)	
	Female	Male
No exposure	848 | 10534.1 | 80.5 (75.2 ; 86.1)	339 | 6948.1 | 48.8 (43.7 ; 54.3)
Exposed	602 | 9996.2 | 60.2 (55.5 ; 65.2)	225 | 6681.2 | 33.7 (29.4 ; 38.4)

ESM Table 14: Estimated Rate Ratios of DKA admissions for CSII-exposed person-time vs pre-package person-time and for pre-package trend from models adjusted for age, sex, diabetes duration and HbA_{1c} at baseline - stratified by sex - Posterior mean (95% Credible Interval) and posterior probability of the direction of the effect (pp)-

CSII Exposure status	Female		Male	
	PM (95% CrI)	pp	PM (95% CrI)	pp
Time effect (years)	1.03 (0.99 ; 1.08)	0.91	1.03 (0.96 ; 1.11)	0.84
Pump-exposure status				
ref= Unexposed				
Exposed	0.63 (0.47 ; 0.84)	1.00	0.53 (0.33 ; 0.86)	0.99

ESM Table 15: Crude DKA event-rates and associated 95% Confidence Intervals by CSII exposure status - Stratified by age band at pump initiation - (E denotes the number of events observed and PY the number of person-years observed)

Pump Exposure status	E|PY| Crude event rate (95% CI)					
	<13 y.o.	13-18 y.o.	19-24 y.o.	25-44 y.o.	45-64 y.o.	>64 y.o.
No exposure	254|2542.8|99.9 (88.0; 113.0)	140|1984.7|70.5 (59.3; 83.2)	344|1380.7|249.1 (223.5; 276.9)	372|7428.4|50.1 (45.1; 55.4)	74|3852.2|19.2 (15.1; 24.1)	E<10
Exposed	132|3871.0|34.1 (28.5; 40.4)	223|2211.8|100.8 (88.0; 115.0)	117|1123.1|104.2 (86.2; 124.8)	246|6064.5|40.6 (35.7; 46.0)	100|3203.4|31.2 (25.4; 38.0)	E<10

ESM Table 16: Estimated Rate Ratios of DKA admissions for CSII-exposed person-time vs pre-package person-time and for pre-package trend from models adjusted for age, sex, diabetes duration and HbA_{1c} at baseline - stratified by age-band at pump initiation Posterior mean (95% Credible Interval) and posterior probability of the direction of the effect (pp))

CSII Exposure status	<13 y.o.		13-18 y.o.		19-24 y.o.		25-44 y.o.		45-64 y.o.		>64 y.o.	
	PM (95% CrI)	pp	PM (95% CrI)	pp	PM (95% CrI)	pp	PM (95% CrI)	pp	PM (95% CrI)	pp	PM (95% CrI)	pp
Time effect (years)	1.03 (0.94 ; 1.13)	0.77	1.08 (0.99 ; 1.18)	0.95	0.99 (0.89 ; 1.10)	0.61	1.02 (0.96 ; 1.09)	0.74	1.03 (0.94 ; 1.12)	0.70	Low sample size	-
Pump-exposure status											-	-
ref= Unexposed											-	-
Exposed	0.23 (0.12 ; 0.44)	1.00	1.05 (0.59 ; 1.89)	0.57	0.45 (0.23 ; 0.85)	0.99	0.63 (0.41 ; 0.96)	0.99	1.50 (0.75 ; 3.05)	0.87	-	-

ESM Table 17: Crude DKA event-rates and associated 95% Confidence Intervals by pump exposure status - Stratified by SIMD quintile - (E denotes the number of events observed and PY the number of person-years observed)

<table>
<tr><th>CSII Exposure status</th><th colspan="5">E|PY| Crude event rate (95% CI)</th></tr>
<tr><th></th><th>Q1 (most deprived).</th><th>Q2</th><th>Q3</th><th>Q4</th><th>Q5 (least deprived)</th></tr>
<tr><td>No exposure</td><td>285 | 2229.0 | 127.9 (113.4 ; 143.6)</td><td>244 | 2987.9 | 81.7 (71.7 ; 92.6)</td><td>248 | 3390.5 | 73.1 (64.3 ; 82.8)</td><td>164 | 3381.8 | 48.5 (41.4 ; 56.5)</td><td>158 | 3803.5 | 41.5 (35.3 ; 48.5)</td></tr>
<tr><td>Exposed</td><td>224 | 2074.0 | 108.0 (94.3 ; 123.1)</td><td>180 | 2596.1 | 69.3 (59.6 ; 80.2)</td><td>138 | 3302.3 | 41.8 (35.1 ; 49.4)</td><td>113 | 3383.0 | 33.4 (27.5 ; 40.2)</td><td>107 | 3758.3 | 28.5 (23.3 ; 34.4)</td></tr>
</table>

ESM Table 18: Estimated Rate Ratios of DKA admissions for CSII-exposed person-time vs pre-package person-time and for pre-package trend from models adjusted for age, sex, diabetes duration and HbA_{1c} at baseline - stratified by SIMD Quintile - Posterior mean (95% Credible Interval) and posterior probability of the direction of the effect (pp)

Pump Exposure status	Q1 (most deprived)		Q2		Q3		Q4		Q5	
	PM (95% CrI)	pp	PM (95% CrI)	pp	PM (95% CrI)	pp	PM (95% CrI)	pp	PM (95% CrI)	pp
Time effect (years)	1.07 (0.99 ; 1.16)	0.96	1.08 (1.00 ; 1.17)	0.97	1.01 (0.93 ; 1.09)	0.58	0.99 (0.90 ; 1.09)	0.54	1.00 (0.91 ; 1.10)	0.53
Pump-exposure status										
ref= Unexposed										
Exposed	0.68 (0.41 ; 1.16)	0.93	0.50 (0.30 ; 0.84)	0.99	0.52 (0.30 ; 0.88)	0.99	0.61 (0.33 ; 1.17)	0.93	0.67 (0.35 ; 1.27)	0.89

ESM Table 19: Crude DKA event-rates and associated 95% Confidence Intervals by CSII exposure status - Stratified by prior FM/CGM usage - (E denotes the number of events observed and PY the number of person-years observed) -

<table>
<tr><th>Pump Exposure status</th><th colspan="2">E|PY| Crude event rate (95% CI)</th></tr>
<tr><th></th><th>No prior FM/CGM usage</th><th>Prior FM/CGM usage</th></tr>
<tr><td>No exposure</td><td>1082 | 16079.5 | 67.3 (63.3 ; 71.4)</td><td>105 | 1402.7 | 74.9 (61.2 ; 90.6)</td></tr>
<tr><td>Exposed</td><td>820 | 16407.3 | 50.0 (46.6 ; 53.5)</td><td>E<10| 270.1 |</td></tr>
</table>

ESM Table 20: Estimated Rate Ratios of DKA admissions for CSII-exposed person-time vs pre-package person-time and for pre-package trend from models adjusted for age, diabetes duration, and HbA_{1c} at baseline - stratified by prior FM/CGM usage -Posterior mean (95% Credible Interval) and posterior probability of the direction of the effect (pp)

CSII Exposure status	No Prior FM/CGM use		Prior FM/CGM use	
	PM (95% CrI)	pp	PM (95% CrI)	pp
Time effect (years)	1.03 (0.99 ; 1.07)	0.94	Low sample size	-
Pump-exposure status			-	-
ref= Unexposed			-	-
Exposed	0.63 (0.49 ; 0.80)	1.00	-	-

ESM Table 21: Estimated Rate Ratios of SHH admissions for CSII-exposed person-time vs pre-package person-time and for pre-package trend from models adjusted for age, sex, diabetes duration, and HbA_{1c} at baseline - Posterior mean (95% Credible Interval) and posterior probability of the direction of the effect (pp)-

Variable	Posterior mean (95% Credible Interval)	pp
Time effect (years)	1.01 (0.95 ; 1.07)	0.65
Pump-exposure status		
ref= Unexposed		
Exposed	0.67 (0.45 ; 1.01)	0.97

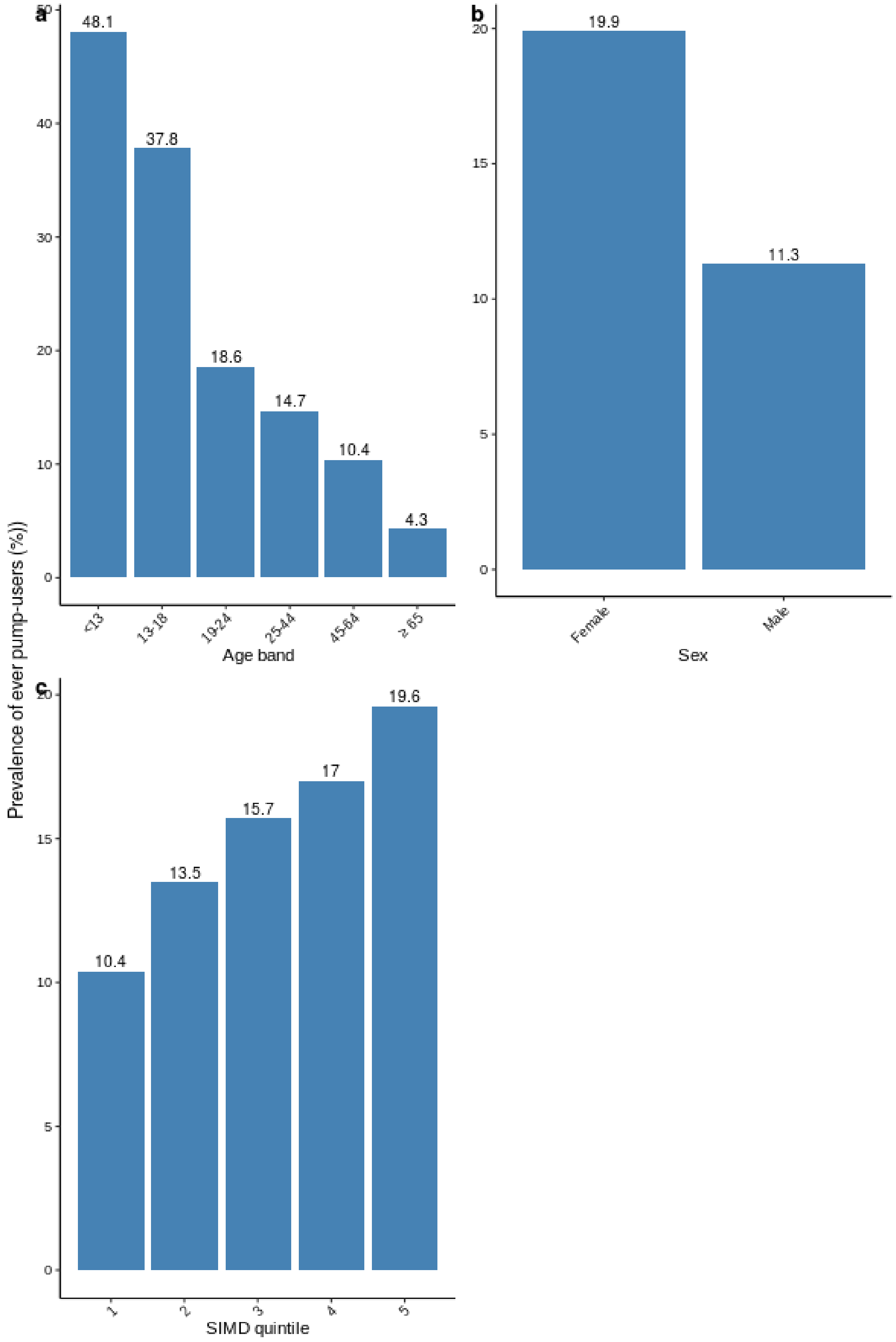

ESM Fig. 1: Current crude prevalence of CSII-usage, stratified by age band at CSII initiation (a), sex (b) and SIMD quintile (c)

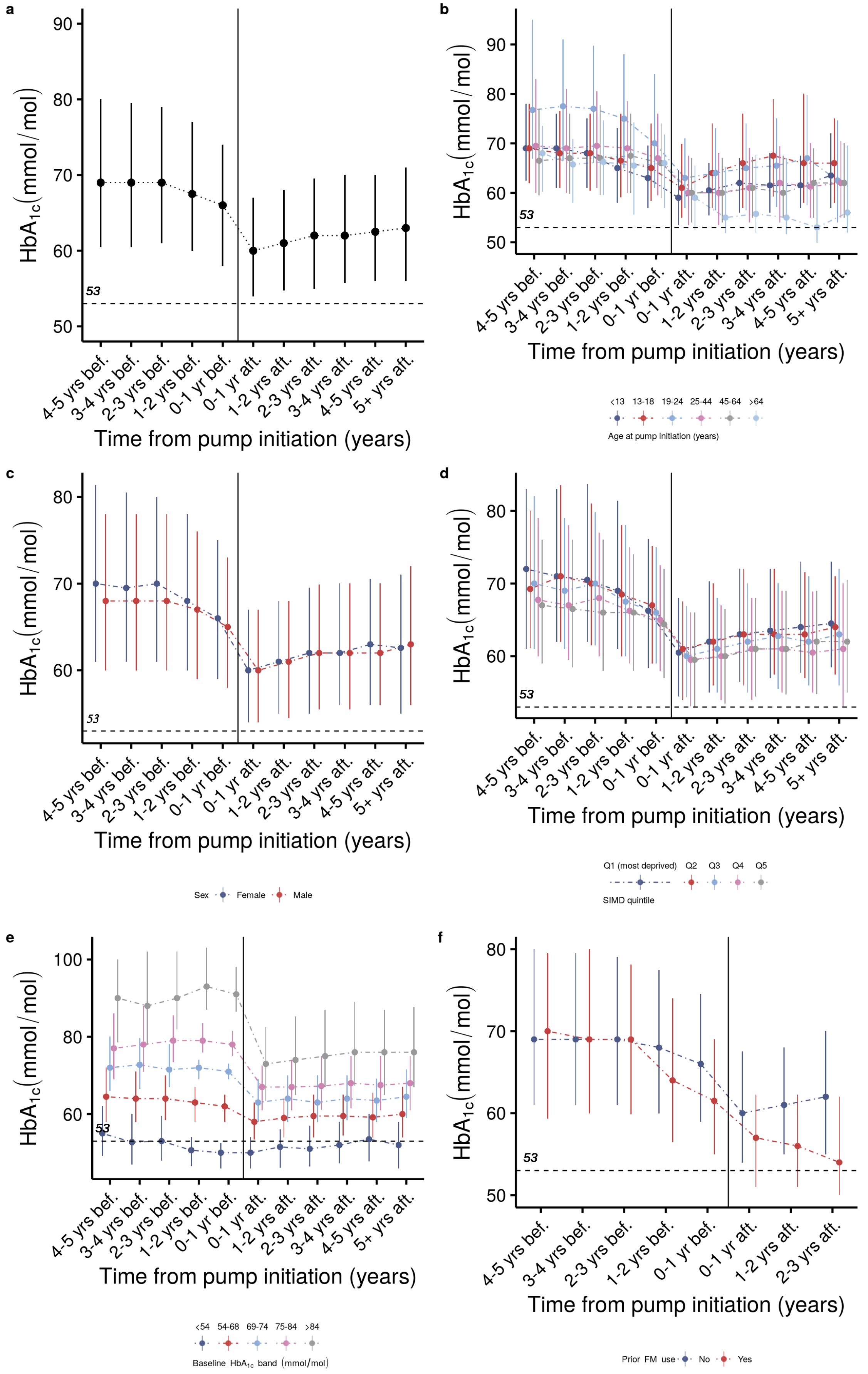

ESM Fig. 2: HbA_{1c} levels (mmol/mol) over time from CSII initiation (years) among CSII users, overall (a) and stratified by age band at CSII initiation (b), sex (c), SIMD quintile (d), baseline HbA_{1c} band (e), prior FM/CGM use (f)

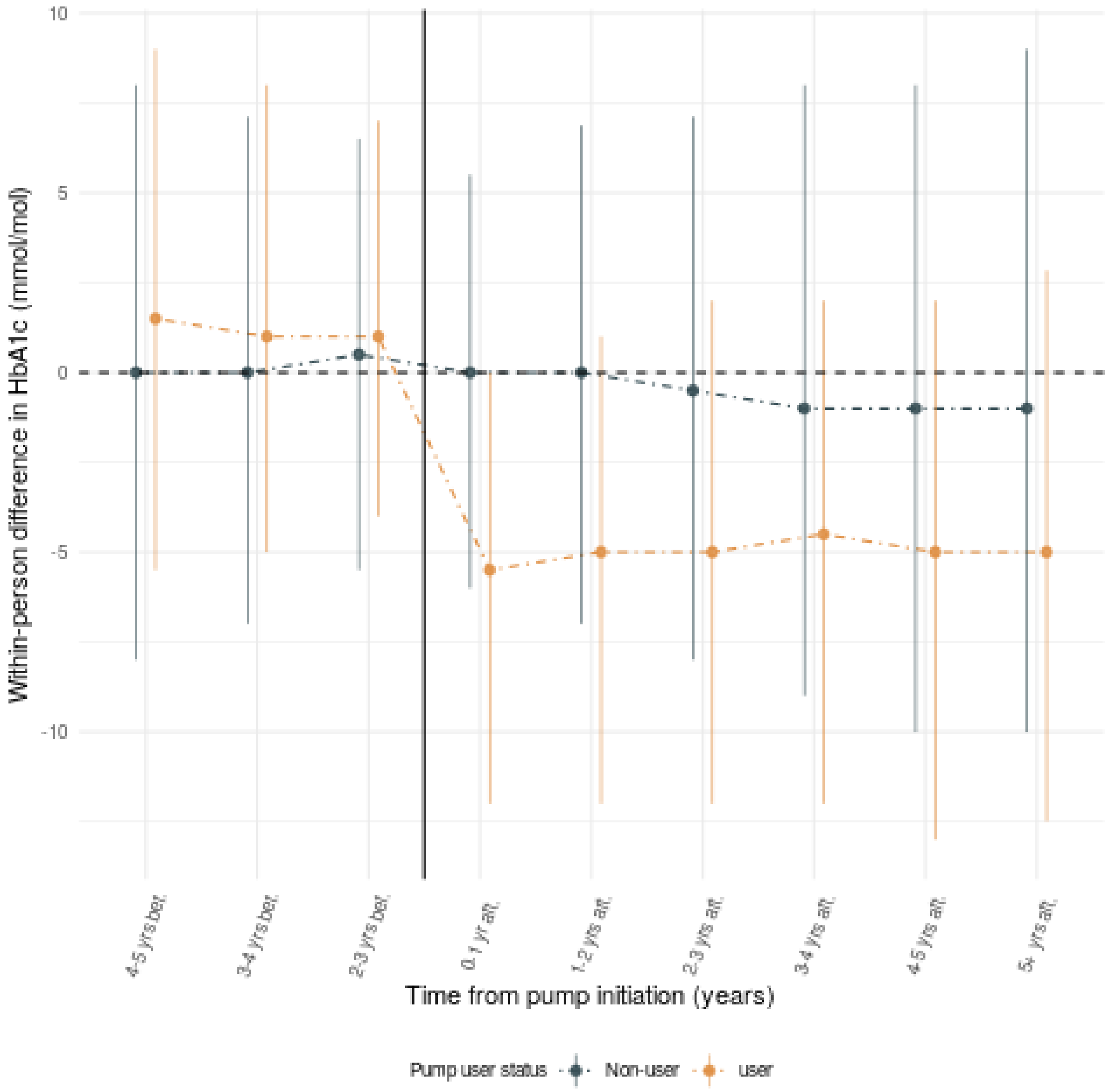

ESM Fig. 3: CSII users vs matched non-users- within-person change from baseline in HbA_{1c} (mmol/mol) over time from CSII initiation date (years)

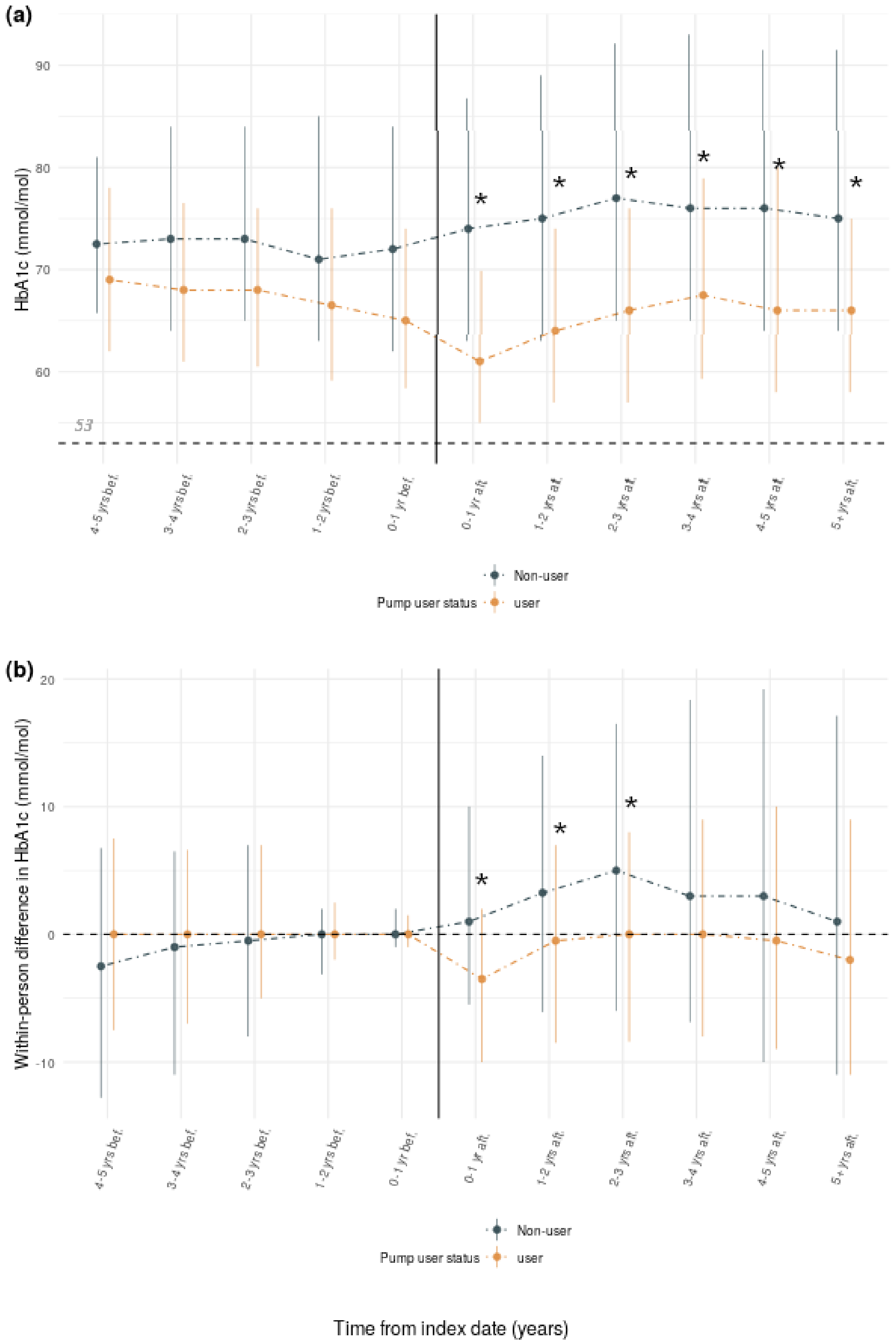

ESM Fig. 4: Adolescents subgroup (13-18y.)- CSII adolescent users versus matched non-users- HbA_{1c} levels (a) and change from baseline in HbA_{1c} (b) (mmol/mol) over time from CSII initiation date (years) - Significance of one-sided signed-rank Wilcoxon test p adjusted for multiple comparisons, of paired user vs matched non-users difference in post-index years denoted by (*)

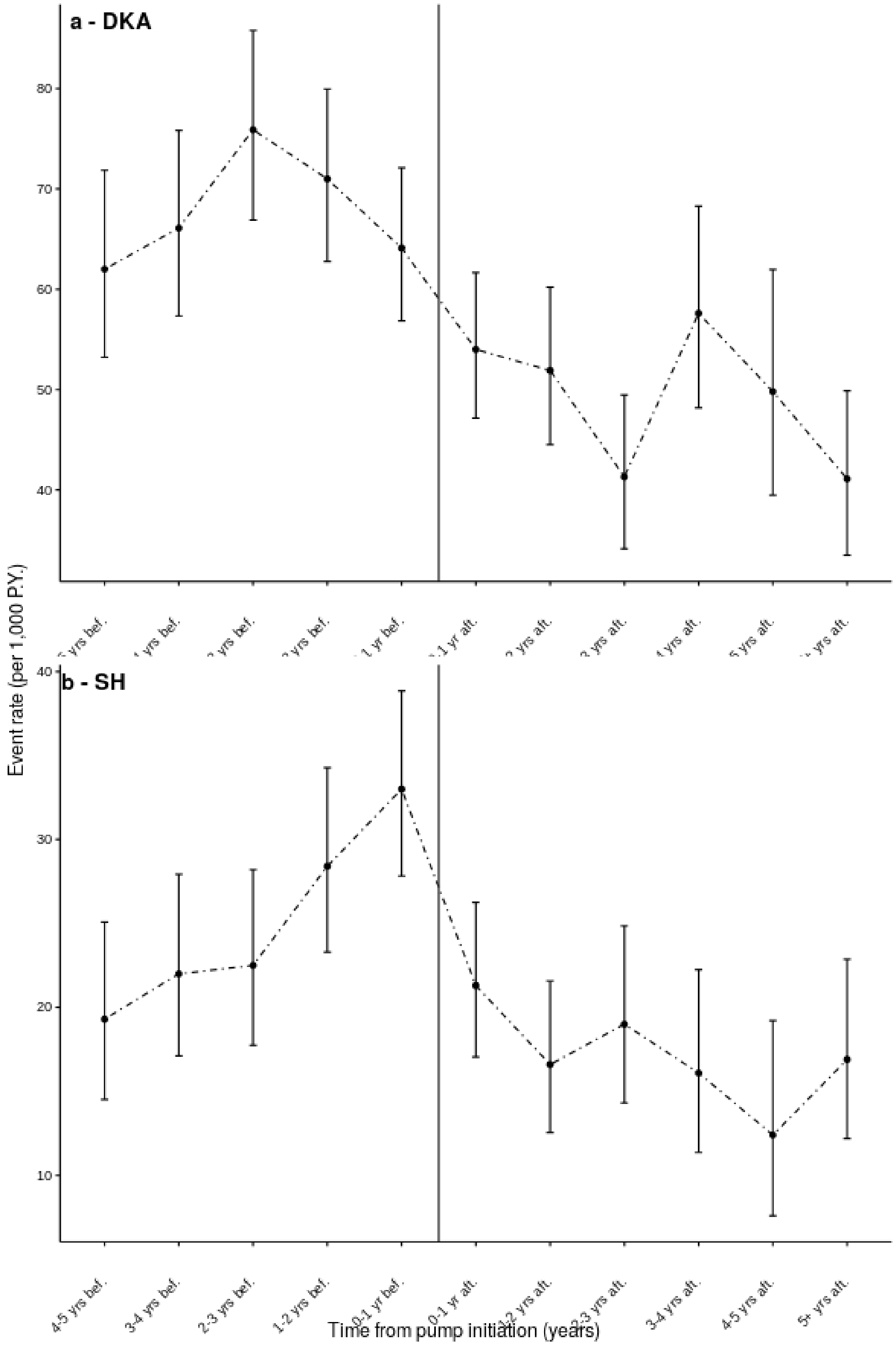

ESM Fig. 5: Crude event-rates and associated 95% Confidence Intervals, by time from CSII initiation (years) - DKA (a) and SHH (b)

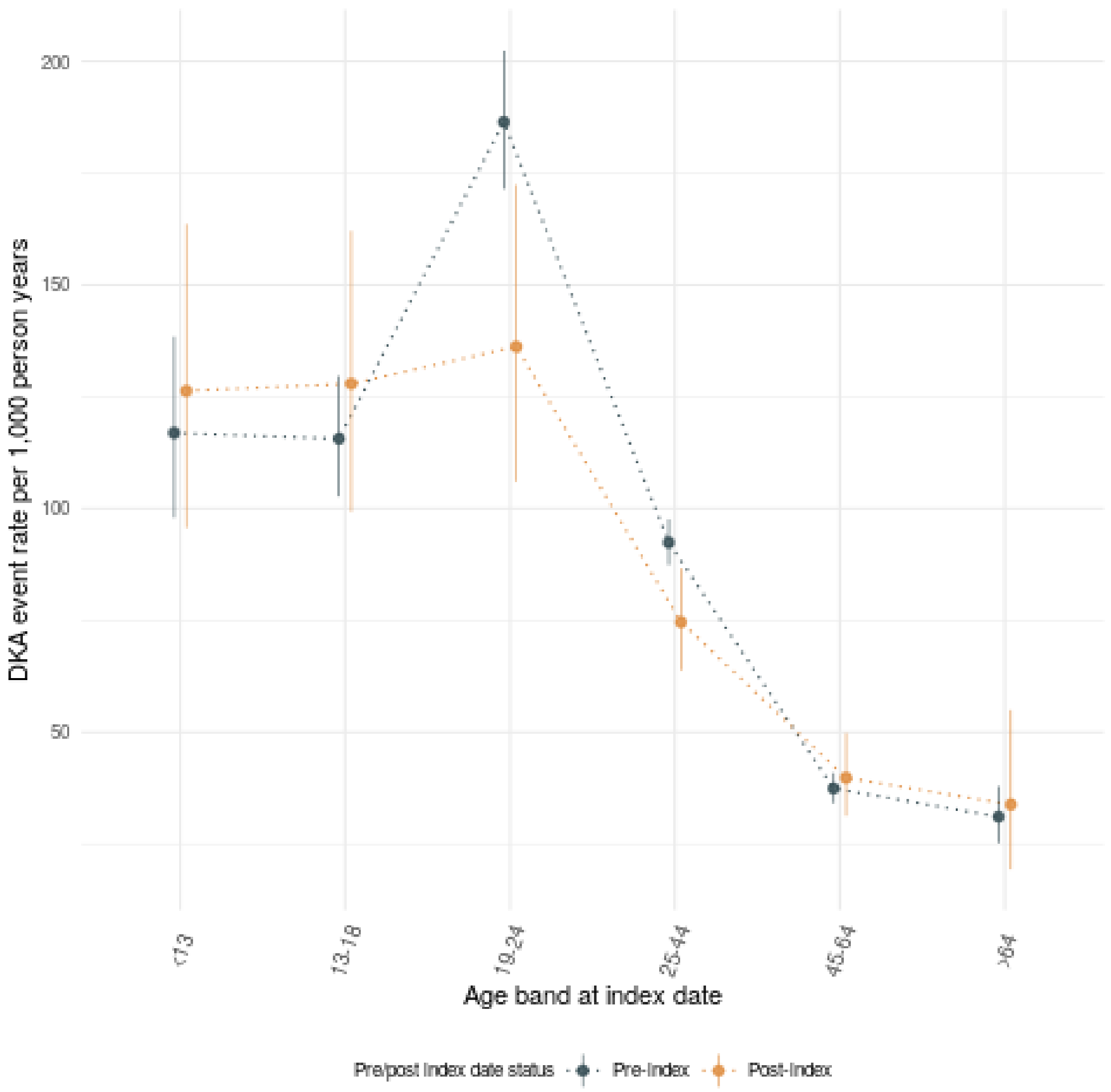

ESM Fig. 6: DKA crude event rates and 95% CI in non-user control group - pre vs post index date- by age band at index date
